# A Soybean bZIP Transcription Factor *GmbZIP19* Confers Multiple Biotic and Abiotic Stress Responses in Plant

**DOI:** 10.3390/ijms21134701

**Published:** 2020-07-01

**Authors:** Qing He, Hanyang Cai, Mengyan Bai, Man Zhang, Fangqian Chen, Youmei Huang, S. V. G. N. Priyadarshani, Mengnan Chai, Liping Liu, Yanhui Liu, Huihuang Chen, Yuan Qin

**Affiliations:** 1 State Key Laboratory of Ecological Pest Control for Fujian and Taiwan Crops, Key Laboratory of Genetics, Breeding and Multiple Utilization of Crops, Ministry of Education, Fujian Provincial Key Laboratory of Haixia Applied Plant Systems Biology, College of Plant Protection, Fujian Agriculture and Forestry University, Fuzhou 350002, China; heqing@fafu.edu.cn (Q.H.); caihanyang123@163.com (H.C.); zhangman3043@163.com (M.Z.); chenfangqian96@163.com (F.C.); hym9995@163.com (Y.H.); niro323@yahoo.com (S.V.G.N.P.); chaimengnan1@163.com (M.C.); Huihuangchen@outlook.com (H.C.); 2 College of Life Science, Fujian Agriculture and Forestry University, Fuzhou 350002, China; mengyanbai@yeah.net (M.B.); 18398636100@163.com (L.L.); yanhuiliu520@gmail.com (Y.L.); 3 State Key Laboratory for Conservation and Utilization of Subtropical Agro-Bioresources, Guangxi Key Lab of Sugarcane Biology, College of Agriculture, Guangxi University, Nanning 530004, China

**Keywords:** *GmbZIP19*, transcription factor, biotic stress, abiotic stress

## Abstract

The basic leucine zipper (bZIP) is a plant-specific transcription factor family that plays crucial roles in response to biotic and abiotic stresses. However, little is known about the function of bZIP genes in soybean. In this study, we isolated a bZIP gene, *GmbZIP19*, from soybean. A subcellular localization study of *GmbZIP19* revealed its nucleus localization. We showed that *GmbZIP19* expression was significantly induced by ABA (abscisic acid), JA (jasmonic acid) and SA (salicylic acid), but reduced under salt and drought stress conditions. Further, *GmbZIP19* overexpression *Arabidopsis* lines showed increased resistance to *S. sclerotiorum* and *Pseudomonas syringae* associated with upregulated ABA-, JA-, ETH- (ethephon-)and SA-induced marker genes expression, but exhibited sensitivity to salt and drought stresses in association with destroyed stomatal closure and downregulated the salt and drought stresses marker genes’ expression. We generated a soybean transient *GmbZIP19* overexpression line, performed a Chromatin immunoprecipitation assay and found that GmbZIP19 bound to promoters of ABA-, JA-, ETH-, and SA-induced marker genes in soybean. The yeast one-hybrid verified the combination. The current study suggested that *GmbZIP19* is a positive regulator of pathogen resistance and a negative regulator of salt and drought stress tolerance.

## 1. Introduction

Soybean, an important economic crop and one of the main oil crops worldwide, is widely used as a source for plant oil and as a protein resource by humans. Extreme environmental conditions severely influence the productivity of soybean and weaken the crop productivity, ultimately affecting global food security [1]. Furthermore, various environmental conditions such as salinity, drought, temperature changes, nutritional deficiency and pathogen invasion significantly influence soybean growth and development, leading to reduced production. Evolutionarily, in order to overcome these stress conditions, plants have developed a series of response mechanisms at the physiological, morphological, cellular, biochemical and molecular levels [2,3,4]. Gene expression changes play an important role during stress response. Transcriptional factors can mediate some stress-related gene expressions and further regulate the responses to adverse stresses in plants [5,6]. One of these is the basic leucine zipper (bZIP) transcription factor.

The basic leucine zipper (bZIP) transcription factor family is one of the largest and the most diverse transcription factor (TF) families in plants. bZIP transcription factors own a highly conserved 40–80 amino acid bZIP domain, which consists of a conserved basic region and a leucine zipper [7,8]. The conserved basic region contains approximate 16 amino acid residues with a N-X7- R/K motif consists of a nuclear localization signal and DNA binding domain, whereas the leucine zipper is less conserved and forms a heptad repeat of leucine residues or other bulky hydrophobic amino acids (Ile, Val, Phe, or Met) [9,10]. It has been indicated that the plant bZIP proteins bind to DNA sequences with an ACGT core cis-element, especially ABRE (ABA-responsive element), G-box (CACGTG), C-box (GACGTC) and A-box (TACGTA) [11].

In plants, it has been indicated that bZIP transcription factors are involved in various biological processes, such as hormone signaling pathways and tolerance to stresses. Firstly, bZIP TFs (transcription factors) are involved in sugar and hormone signaling. For instance, a crucial repressor for ethylene biosynthesis in *Arabidopsis*, *AtERF11*, is modulated by the bZIP transcription factor HY5 [12]. In addition, previous studies have shown that bZIP transcription factors participate in the responses to biotic and abiotic stresses such as salinity, drought, cold and pathogen infections. *TabZIP60*, a novel wheat bZIP transcription factor, confers multiple abiotic stress tolerances in transgenic *Arabidopsis* [13]. bZIP transcription factors have been identified and studied in different kinds of plant species due to their high value in plant development and stress tolerance. 89 bZIPs were identified in *Oryza sativa* (rice) [14], 75 bZIP genes have been identified in *Arabidopsis thaliana* [8], 64 in cucumber [15], 125 in *Zea mays* (maize) [16] and 160 in *Glycine max* (soybean) [17].

Although there are several conserved and novel bZIP genes identified in different processes during soybean development, *GmFT2a* and *GmFT5a* redundantly and differentially regulate flowering through interaction with and upregulation of the bZIP transcription factor *GmFDL19* in soybean [18], overexpression of *GmFDL19* enhances tolerance to drought and salt stresses in soybean [19] and *GmbZIP2* confers drought and salt resistances in transgenic *Arabidopsis* and soybean [20]. In general, soybean bZIP genes were seldom studied in soybean, especially in stress responses. Previously, studies have shown that *AtbZIP19* is essential for *Arabidopsis* adaptation to Zn deficiency in roots [21] and it can interact with *AtbZIP23* to regulate the adaptation [22]. There are no studies that showed the stress responses of *AtbZIP19*. In the current study, *GmbZIP19*, a homologous gene of *AtbZIP19* in soybean, was identified from a full-length soybean cDNA bank. Expression profile indicated that the expression of *GmbZIP19* was induced by *S. sclerotiorum*, and exogenous hormones like JA (jasmonic acid), SA (salicylic acid), ETH (ethephon), ABA (abscisic acid) and BR (brassinolide) but reduced by salinity and drought. Overexpression of *GmbZIP19* in *Arabidopsis* showed more resistance to *S. sclerotiorum* and *Pseudomonas syringae*, but more sensitivity to salinity, drought and different hormones, including ABA, JA and ETH, compared with wild-type (WT) plants. In summary, our results verified that *GmbZIP19* plays an important role in multiple abiotic and biotic stress responses.

## 2. Results

### 2.1. Basic Bioinformatic Analyses of GmbZIP19

According to https://web.expasy.org, *GmbZIP19* cDNA was predicted to be 1617 bp long, containing a 723-bp ORF (open reading frame), which encodes a polypeptide of 240 amino acids with a predicted molecular weight of 26.57 kDa and a theoretical pI of 6.59. Sequence alignment showed high levels of amino acid sequence similarity of *GmbZIP19* with three other bZIP proteins from *Arabidopsis* and *Oryza sativa* (rice) (Figure S1). They shared a conserved bZIP DNA-binding domain, a basic DNA binding region and a leucine zipper dimerization motif. The basic DNA binding region is conserved and contains a 52-amino acid long basic region (N-x7-R/K-x9).

### 2.2. Subcellular Localization of GmbZIP19

To determine the subcellular localization of GmbZIP19 protein, the *GmbZIP19* CDS was fused to the pGWB605-GFP vector. The recombinant vector (35S-*GmbZIP19*-GFP) and the 35S promoter-driven GFP control vector (35S-GFP) were transiently expressed in *N. benthamiana* leaves through *Agrobacterium* infection. As shown in Figure S2, the 35S-GFP control was observed in both the nucleus and cytoplasm membrane, whereas 35S-*GmbZIP19*-GFP was specifically localized in the nucleus.

### 2.3. Expression Profile of GmbZIP19 in Response to Biotic and Abiotic Stresses

**Figure 1:**
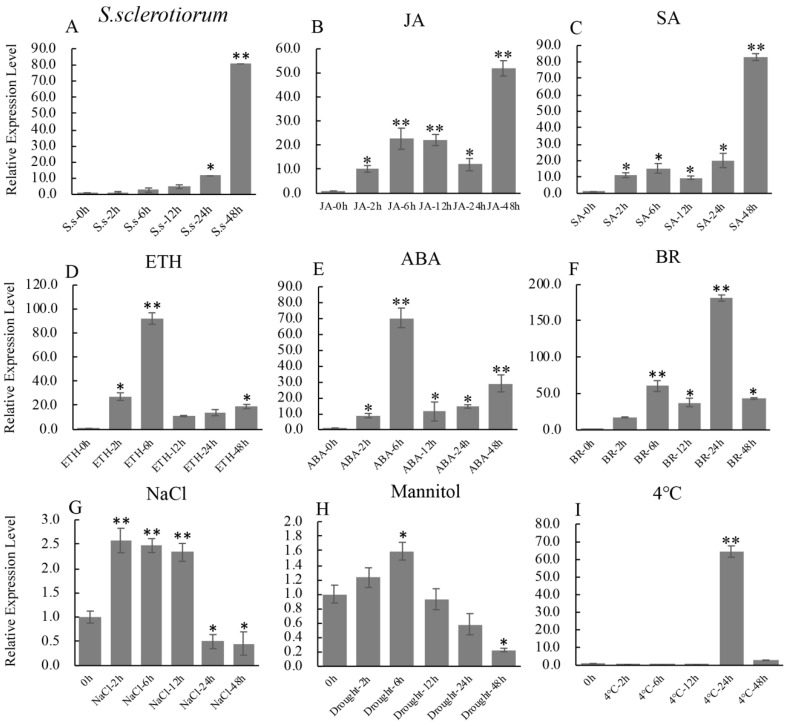
The expression profile of *GmbZIP19* in response to biotic and abiotic stresses. (**A**–**I**) The expression profile of *GmbZIP19* in response to *S. sclerotiorum*, 100 μM JA (jasmonic acid), 1 mM SA (salicylic acid), 100 μM ABA (abscisic acid), 1 mM ETH (ethephon), 100 μM BR (brassinolide), 100 mM NaCl for salt condition, 250 mM Mannitol for drought condition and 4 °C for low temperature. Three technical replicates were performed for each of the three independent biological replicates. The error bars were obtained from multiple replicates of the RT-qPCR and indicate ±SD (*n* = 3 replicates). Asterisks indicate significant differences for the indicated comparisons based on a Student’s *t*-test (** *p* < 0.01; * *p* < 0.05).

Bioinformatics analysis indicated that there are many predicted stress response-related cis-elements: G-box recognition site (1 hit), MYB recognition site (6 hits), CGTCA motif (1 hit) and TCA-element (3 hits) in the promoter of *GmbZIP19* (Figure S3), suggesting that *GmbZIP19* may be involved in biotic and abiotic stress responses. In order to further explore and predict the function of *GmbZIP19*, the expression changes of *GmbZIP19* in soybean leaves under different biotic and abiotic treatments was evaluated by RT-qPCR. The expression of *GmbZIP19* was obviously induced by *S. sclerotiorum*, a typical necrotrophic phytopathogen in soybean [23] (Figure 1A).

Previous studies have shown that plant defense to pathogen was associated with the mediation of various plant hormones, including SA, JA, ABA and ETH. Therefore, we examined the expression of *GmbZIP19* under different hormones. The result showed that *GmbZIP19* expression was induced rapidly by JA, SA, ETH, ABA and BR within 2 h after treatment (Figure 1B–F). *GmbZIP19* maintained gradually increased expression under JA and SA treatments (Figure 1B,C) while the expression level of *GmbZIP19* in response to ETH and ABA peaked at 6 h and decreased rapidly (Figure 1D,E). These results indicated that the expression of *GmbZIP19* may be related to the defense responses mediated by JA, SA, ETH and ABA in different manners.

In addition, the expression of *GmbZIP19* was significantly induced by NaCl at 2, 6 and 12 h, but significantly reduced at 24 and 48 h (Figure 1G). The expression of *GmbZIP19* was also induced by mannitol (drought) treatment and reached to the peak at 6 h, and then gradually decreased and reached to the lowest level at 48 h after treatment (Figure 1H), while cold (4 °C) induced the expression of *GmbZIP19* at 24 h (Figure 1I).

**Figure 2:**
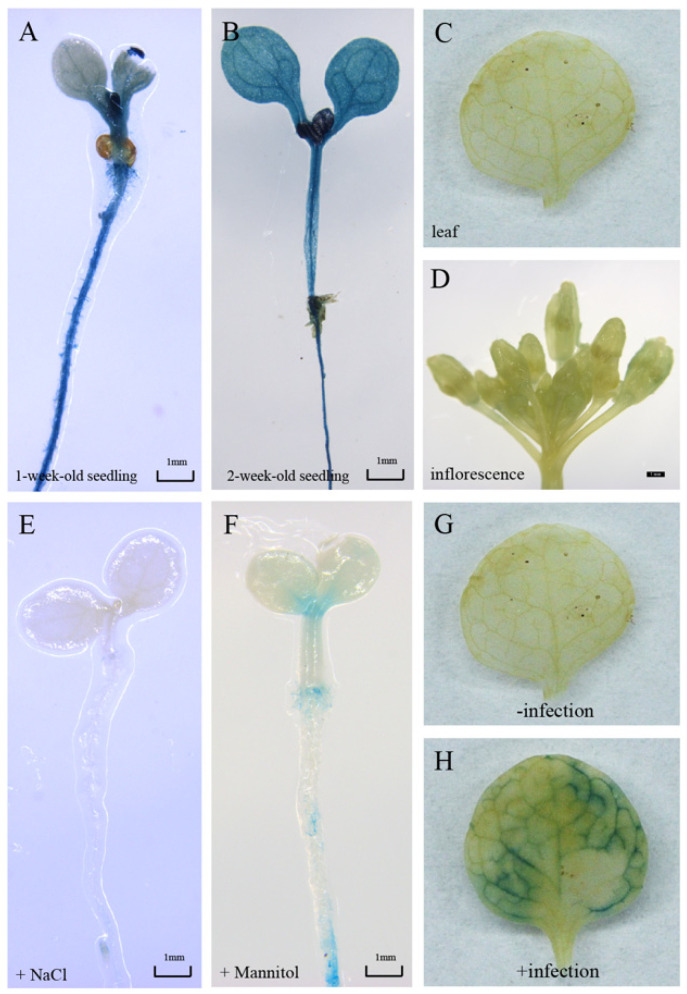
The responses of the *GmbZIP19* promoter to salt, drought and *S. sclerotiorum*. (**A**) GUS staining result of 1-week-old *pGmbZIP19-GUS Arabidopsis* seedlings. (**B**) GUS staining result of two-week-old *pGmbZIP19-GUS Arabidopsis* seedlings. Bar = 1 mm. (**C**) GUS staining result of 4-week-old *pGmbZIP19-GUS Arabidopsis* leaf. (**D**) GUS staining result of 6-week-old *pGmbZIP19-GUS Arabidopsis* inflorescence. Bar = 1 mm. (**E**) GUS staining result of 1-week-old *pGmbZIP19-GUS Arabidopsis* seedlings under 150 mM NaCl for 6 days. Bar = 1 mm. (**F**) GUS staining result of 1-week-old *pGmbZIP19-GUS Arabidopsis* seedlings under 300 mM mannitol for 6 days. Bar = 1 mm. (**G**) GUS staining result of *pGmbZIP19-GUS Arabidopsis* leaf under 0 h *S. scleroterium* infection. (**H**) GUS staining result of *pGmbZIP19-GUS Arabidopsis* leaf under 6 h *S. scleroterium* infection.

In order to verify the expression pattern of *GmbZIP19*, we generated *pGmbZIP19-GUS* fusion transgenic *Arabidopsis* plants (*pGmbZIP19-GUS*). GUS staining was detected in the shoot apical meristem (SAM), hypocotyl and root of one-week-old *pGmbZIP19-GUS* seedlings (Figure 2A). A similar GUS expression pattern was also examined in two-week-old *pGmbZIP19-GUS* seedlings (Figure 2B) but no GUS signal was found in mature *pGmbZIP19-GUS* plants, including in the leaves and inflorescence (Figure 2C,D). Interestingly, the *pGmbZIP19-GUS* signal was significantly reduced when one-week-old *pGmbZIP19-GUS* seedlings were treated with 150 mM NaCl or 300 mM mannitol for 7 days (Figure 2E,F), suggesting that the expression of *GmbZIP19* was inhibited by salt and drought. These results were consistent with the reduced expression of *GmbZIP19* evaluated by RT-qPCR after salt and drought (mimic using mannitol) treatment after 24 h (Figure 1), which indicated that *GmbZIP19* may be involved in regulating different abiotic stress tolerance.

In addition, we compared the GUS expression in the leaf of mature *pGmbZIP19-GUS* plants before and after inoculation of *S. sclerotiorum.* In line with the induced expression of *GmbZIP19* in plants after *S. sclerotiorum* inoculation (Figure 1A), an increased GUS signal was detected in *pGmbZIP19-GUS* plants after inoculation of *S. sclerotiorum* (Figure 2G,H). These results indicated that *GmbZIP19* was induced by this pathogen and it may be involved in the pathogen defense process.

### 2.4. Overexpression of GmbZIP19 in Arabidopsis Increases the Tolerance to Pathogen

**Figure 3:**
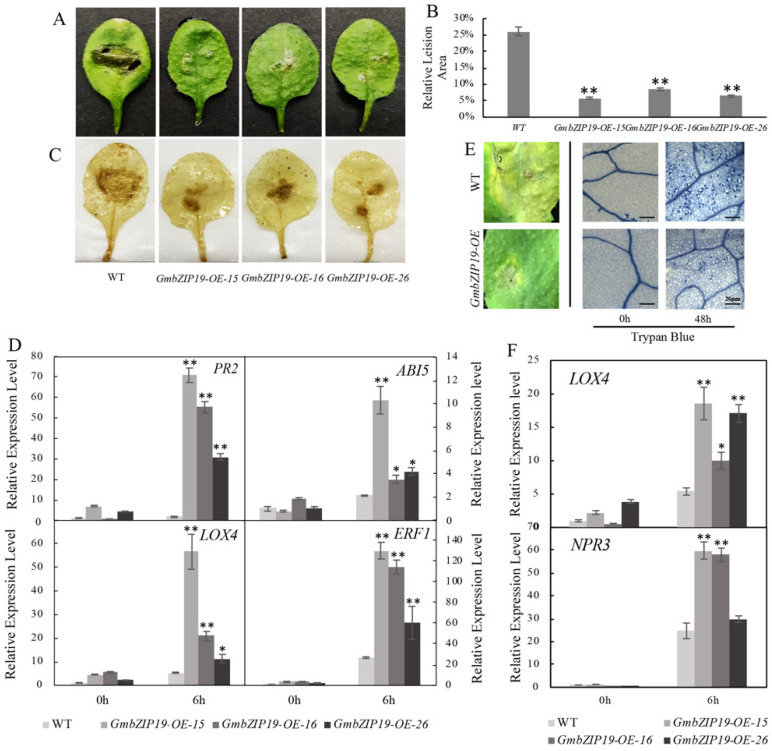
Overexpression of *GmbZIP19* confers improved disease resistance against *S. sclerotiorum* and *Pseudomonas syringae*. (**A**) The phenotype of *GmbZIP19-OE* lines and WT after *S. scleroterium* infection. (**B**) The relative lesion area of *GmbZIP19-OE* lines and WT under *S. scleroterium* infection. Error bars indicate ± SD (*n* = 5 leaves). (**C**) The DAB staining result (accumulation of H_2_O_2_ in leaves) of *GmbZIP19-OE* lines and WT after *S. scleroterium* infection. (**D**) qPCR analysis of transcription levels in *GmbZIP19-OE* transgenic and WT plants after *S. scleroterium* infection. (**E**) The phenotype and trypan blue staining result of GmbZIP19-OE and WT plants under *Pseudomonas syringae* infection. Bar = 20 μm. (**F**) qPCR analysis of transcription levels in *GmbZIP19-OE* transgenic and WT plants after *pst.* DC3000 infection. The error bars were obtained from multiple replicates of the RT-qPCR and indicate ±SD (*n* = 3 replicates). Asterisks indicate significant differences for the indicated comparisons based on a Students’ *t*-test (** *p* < 0.01; * *p* < 0.05).

In order to determine the function of *GmbZIP19* in response to pathogen, we generated 35S promoter-driven overexpression of *GmbZIP19 Arabidopsis* plants (*GmbZIP19-OE*) and inoculated the same size of *S.sclerotiorum*, a typical pathogen that can cause considerable damage and severe production loss in soybean [23], on the surface of leaves of WT and *GmbZIP19-OE* plants. The result showed that the relative lesion areas of three different *GmbZIP19-OE* lines were much smaller than WT at 12 h post-inoculation (hpi) (Figure 3A,B), suggesting that *GmbZIP19-OE* plants exhibited stronger resistance to *S. sclerotiorum* than WT. DAB staining showed that brown precipitates, representing accumulation of H_2_O_2,_ in leaves of *GmbZIP19-OE* lines after infection, was lighter than that in WT (Figure 3C). The result indicates that overexpression of *GmbZIP19* in *Arabidopsis* significantly improved plant tolerance to *S. sclerotiorum.*

To evaluate the possible role of plant hormones in the resistance to *S. sclerotiorum* conferred by *GmbZIP19* in *Arabidopsis*, the relative expression levels of stress-responsive genes *AtPR2*, *AtABI5*, *AtLOX4* and *AtERF1* in association with SA, ABA, JA and ETH respectively, were examined by RT-qPCR. The expression of these four marker genes in *GmbZIP19-OE* plants has almost no significant difference with WT when there is no treatment. In contrast, at 6 hpi to *S. sclerotiorum*, the relative expression levels of these four genes in three *GmbZIP19-OE* transgenic lines significantly increased compared to WT (Figure 3D). Induced expression of SA-, ABA-, JA- and ETH-dependent genes in *GmbZIP19-OE* upon *S. sclerotiorum* inoculation suggested the involvement of these plant hormones in the regulation of *GmbZIP19* to *S. sclerotiorum* resistance.

Furthermore, we investigated whether *GmbZIP19-OE* in *Arabidopsis* can also enhance plant tolerance to *Pseudomonas syringae* (*pst* DC3000), a (hemi) biotrophic bacterial pathogen that can cause bacterial leaf spot disease [24]. After 48 h of *Pseudomonas syringae* infection, WT plants exhibited extremely severe chlorosis symptoms showing large disease spots and a strong trypan blue staining signal, representing the large number of dead cells, in the whole leaf. Nevertheless, the leaves of *GmbZIP19-OE* lines had smaller disease spots and a lighter trypan blue staining signal after *Pseudomonas syringae* inoculation (Figure 3E). The result demonstrated that *GmbZIP19* promotes plant tolerance to *Pseudomonas syringae* as well. Moreover, the increased transcription levels of JA-related gene, *AtLOX4*, and SA-related gene, *AtNPR3*, confirmed the phenotype and suggested that JA- and SA-dependent genes are involved in the regulation of *GmbZIP19* to *pst.* DC3000 (Figure 3F).

### 2.5. Overexpression GmbZIP19 Arabidopsis Exhibits Sensitivity to Salinity

**Figure 4:**
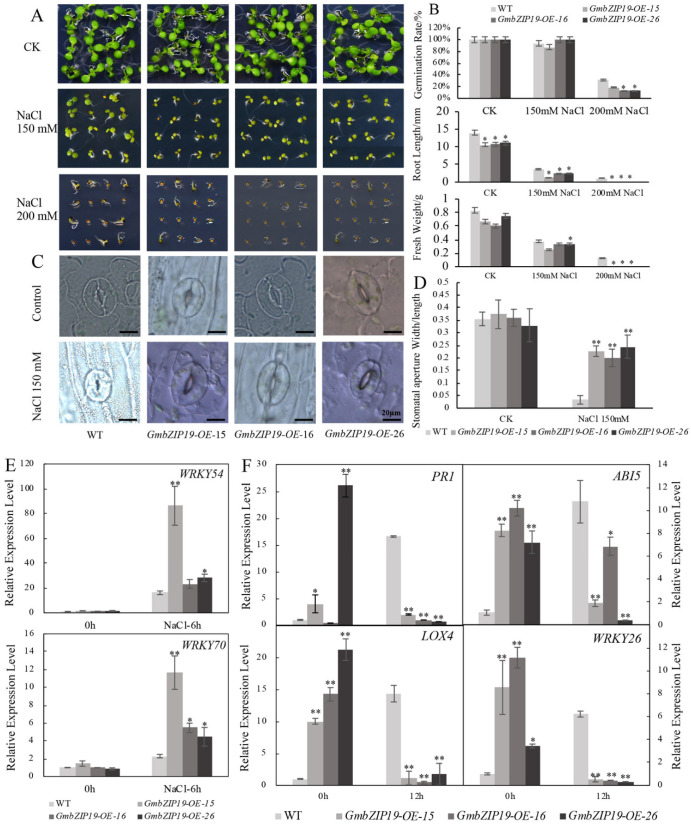
*GmbZIP19*-overexpressed plants display sensitivity to salinity. (**A**) The phenotype of *GmbZIP19-OE* lines and the WT under 150 mM and 200 mM NaCl. (**B**) Quantification of the germination rate, root length and fresh weight of *GmbZIP19-OE* lines and the WT under 150 mM and 200 mM NaCl. The error bars indicate ±SD (*n* > 10 seedlings). (**C**) The stomatal aperture phenotype of *GmbZIP19-OE* lines and WT under 150 mM NaCl. Bar = 20 μm. (**D**) The quantitative analysis of stomatal aperture of *GmbZIP19-OE* lines and WT under 150 mM NaCl. (**E**) The expression levels of stomata movement marker genes in *GmbZIP19-OE* lines and WT under 150 mM NaCl. (**F**) The transcription levels of stress-related genes in *GmbZIP19-OE* lines and the WT under 150 mM NaCl. Three technical replicates were performed for each of the three independent biological replicates. The error bars indicate ±SD (*n* = 3 replicates). Asterisks indicate significant differences for the indicated comparisons based on a Student’s *t*-test (** *p* < 0.01; * *p* < 0.05).

In order to determine the function of *GmbZIP19* in response to salinity in plants, seeds of *GmbZIP19-OE* lines and WT were planted in 1/2 MS plates containing 0, 150 and 200 mM NaCl, respectively. As is shown in Figure 4A, under normal condition (no treatment), there was no obvious difference in growth between WT and *GmbZIP19-OE* plants. However, when exposed to 150 mM and 200 mM NaCl, a continuous decline of root length and fresh weight in both WT and *GmbZIP19-OE* plants was observed with the increasing of salt concentration, but the seed germination only dropped significantly under 150 mM NaCl treatment (Figure 4A,B). Although seed germination showed no significant difference between the WT and *GmbZIP19-OE* lines under control (non-salinity) and 150 mM NaCl, the seed germination rate was declined in *GmbZIP19-OE* lines compared to that in WT when subjected to 200 mM NaCl treatment (Figure 4B). The root length and fresh weight were obviously decreased in *GmbZIP19-OE* lines compared with WT treated with increasing NaCl concentrates (Figure 4A,B). These results indicated that overexpression of *GmbZIP19* in *Arabidopsis* resulted in plant sensitivity to salinity.

With the treatment of 150 mM NaCl, the majority of stomata of WT leaves were completely closed, while the stomata of the *GmbZIP19-OE* lines did not close completely (Figure 4C). The stomatal aperture (width/length) of *GmbZIP19-OE* was extremely higher than that in WT under salt treatment, while there was nearly no difference under the control condition (Figure 4D). The expression levels of *WRKY54* and *WRKY70*, two marker genes negatively regulating the stomatal closure [25], increased significantly in *GmbZIP19-OE* lines compared with that in WT under 150 mM NaCl treatment, while the expression levels of these two genes showed no significant difference in *GmbZIP19-OE* lines and WT under control also confirmed that the stomata of *GmbZIP19-OE* plants was defected. The defective stomatal aperture may be associated with the reduced tolerance of *GmbZIP19-OE* plants to salt.

To evaluate the involvement of plant hormones in the sensitivity of *GmbZIP19-OE* lines to salt, the relative expression levels of stress-responsive genes *AtPR1*, *AtABI5*, *AtLOX4* and *AtWRKY26* related to SA, ABA, JA and abiotic stress respectively, were determined by RT-qPCR. The results showed that the expression of these genes was higher in *GmbZIP19-OE* plants than WT at the normal condition. However, the expression levels of these four genes decreased dramatically in *GmbZIP19-OE* plants in contrast to the significantly increased expression in WT when subjected to 150 mM NaCl treatment stress for 12 h (Figure 4F). These results together indicated that *Arabidopsis* plants overexpressing *GmbZIP19* were less tolerant to salt stress than WT plants and implied the involvement of plant hormone in salinity tolerance.

### 2.6. Overexpression GmbZIP19 Arabidopsis Exhibits Sensitivity to Drought (osmotic stress)

**Figure 5:**
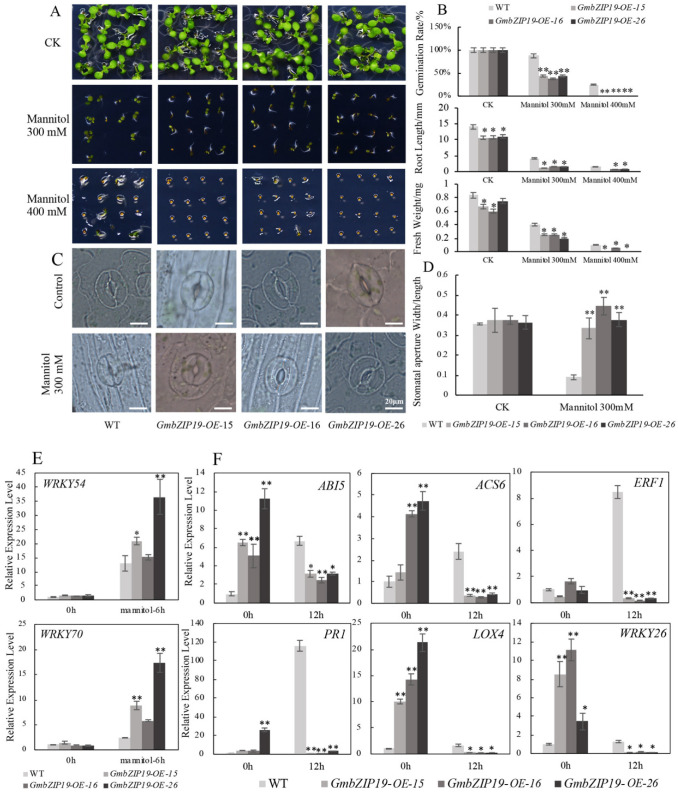
*GmbZIP19*-overexpressed plants display sensitivity to drought. (**A**) The phenotype of *GmbZIP19-OE* transgenic lines and WT under 300 and 400 mM mannitol. (**B**) Quantification of the germination rate, root length and fresh weight of *GmbZIP19-OE* transgenic lines and WT under 300 and 400 mM mannitol. The error bars indicate ±SD (*n* > 10 seedlings). (**C**) The stomatal aperture phenotype of *GmbZIP19-OE* transgenic lines and WT under 300 mM Mannitol. Bar = 20 μm. (**D**) The quantitative analysis of stomatal aperture of *GmbZIP19-OE* transgenic lines and WT under 300 mannitol. (**E**) The expression levels of stomata movement marker genes in *GmbZIP19-OE* lines and WT under 300 mM mannitol. (**F**) The transcription levels of stress-related genes in *GmbZIP19-OE* lines and WT under 300 mM Mannitol. The error bars indicate ±SD (*n* = 3 replicates). Asterisks indicate significant differences for the indicated comparisons based on a Student’s *t*-test (** *p* < 0.01; * *p* < 0.05).

To further analyze the functions of *GmbZIP19* in plant response to osmotic stress, WT and three *GmbZIP19-OE Arabidopsis* lines were subjected to 0, 300 and 400 mM mannitol. WT and *GmbZIP19-OE* plants have no significant difference in germination rate root length and fresh weight when grown in half strength MS medium without mannitol (Figure 5A,B). However, when subjected to drought stress with 300 and 400 mM mannitol, a considerable reduction in seed germination, root length and fresh weight were observed in both *GmbZIP19-OE* and WT plants, and the reduction rate was more severe in *GmbZIP19-OE* lines than WT (Figure 5A,B). These results suggested that overexpression of *GmbZIP19* in *Arabidopsis* reduced plant drought tolerance. The reduced drought tolerance in *GmbZIP19-OE* plants was associated with defects in stomatal closure (Figure 5C–E), suggesting that stomatal closure may contribute to resistance to drought.

Moreover, we examined the relative expression levels of stress-responsive marker genes *AtABI5*, *AtACS6*, *AtLOX4*, *AtERF1*, *AtPR1* and *AtWRKY26* related to ABA (*AtABI5*, *AtACS6*), JA (*AtLOX4*), ETH (*AtERF1*), SA (*AtPR1*) and abiotic stress (*AtWRKY26*) respectively, and found that the expression of these marker genes in *GmbZIP19-OE* plants declined significantly after 12 h drought stress treatment. In contrast, increased marker genes’ expression was detected in WT upon drought stress (Figure 5F). These data together indicated that *GmbZIP19* overexpression caused drought sensitivity in transgenic *Arabidopsis*.

### 2.7. Overexpression GmbZIP19 Arabidopsis Exhibits Sensitivity to Hormones

To further assess the response of *GmbZIP19* to plant hormone, we treated the *GmbZIP19-OE* and WT plants with 450 μM ETH, 200 μM JA and 2.5 μM ABA, respectively. Comparable seed germination rate, root length and fresh weight were examined between the *GmbZIP19-OE* lines and WT grown on MS medium free of exogenous hormones (Figure S4B). The seed germination rate showed no obvious difference upon ETH and JA treatment compared to WT but reduced with JA supplement compared to WT (Figure S4B). Significantly reduced root length and fresh weight were detected in *GmbZIP19-OE* lines compared with WT when they were grown on the medium supplemented with ETH, JA and ABA (Figure S4B). These results showed that *GmbZIP19-OE* lines were sensitive to plant hormones.

### 2.8. Consistent Effects on the Expression of Biotic and Abiotic Stress-Related Genes by Transient Overexpression of GmbZIP19 in Soybean

**Figure 6:**
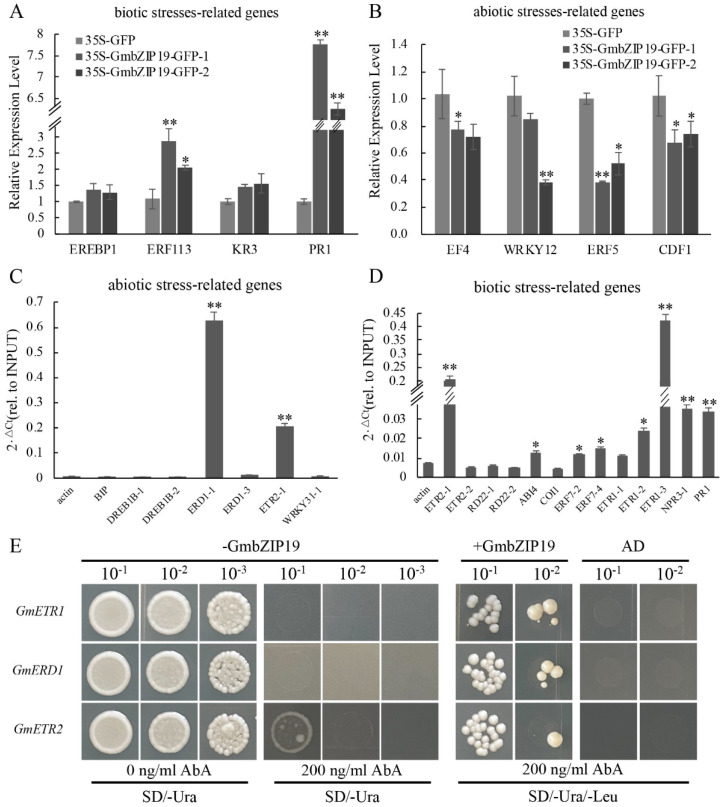
The ChIP result of 35S-*GmbZIP19*–GFP transient expressing soybean. (**A**) qRT-PCR analysis of biotic stress-related genes in 35S-*GmbZIP19*–GFP transient expressing soybean. (**B**) qRT-PCR analysis of abiotic stress-related genes in 35S-*GmbZIP19*–GFP transient expressing soybean. (**C**) ChIP-qPCR analysis of *GmbZIP19* binding to abiotic stress-related genes using GFP antibody and 35S-*GmbZIP19*–GFP transient expressing soybean. (**D**) ChIP-qPCR analysis of *GmbZIP19* binding to biotic stress-related genes using GFP antibody and 35S-*GmbZIP19*–GFP transient expressing soybean. Three independent biological replicates were performed. The error bars indicate ± SD (*n* = 3 replicates). Asterisks indicate significant differences for the indicated comparisons based on a Student’s *t*-test (** *p* < 0.01; * *p* < 0.05). (**E**) The yeast one-hybrid result of GmbZIP19. Three independent biological replicates were performed.

In order to infer the potential role of *GmbZIP19* in response to biotic and abiotic stresses in soybean, we transiently overexpressed *GmbZIP19-GFP* fusion driven by 35S promoter (35S-*GmbZIP19-GFP*) in soybean leaf and evaluated the relative expression levels of biotic and abiotic stress-related genes in the 35S-*GmbZIP19-GFP* and 35S-*GFP* control soybean leaf. As shown in Figure 6A, the relative expression levels of biotic stress-related genes including *EREBP1, ERF113, KR3* and *PR1* were higher in 35S-*GmbZIP19-GFP* soybean leaf than that in the 35S-GFP control, coinciding with the activated stress-related gene expression in *GmbZIP19-OE Arabidopsis* plants upon pathogen infection (Figure 3). These results suggested that the activated biotic stress response was induced by transient overexpression of *GmbZIP19* in soybean. In addition, similar to the decreased expression of the stress-responsive genes in *GmbZIP19-OE Arabidopsis* plants upon salt and drought stresses (Figure 4 and Figure 5), reduced expression of abiotic stress-related genes including *EF4, WRKY12, ERF5* and *CDF1* was examined in 35S-*GmbZIP19-GFP* soybean leaf as compared to 35S-GFP leaf (Figure 6B). These results suggested that transient overexpression of *GmbZIP19* in soybean may also cause enhanced sensitivity to abiotic stress as it does in *Arabidopsis*.

### 2.9. Identification of GmbZIP19 Target Genes

To identify the possible target genes of *GmbZIP19*, Chromatin immunoprecipitation (ChIP) was performed by using *35S-GmbZIP19*-GFP transient expressing soybean. The ChIP assay was performed by using a GFP antibody to pull down the putative *GmbZIP19*-bound DNA from the leaf tissues of 35S-*GmbZIP19*-GFP transient expressing soybean. ChIP-qPCR showed enrichment of *GmbZIP19* in the promoters of some abiotic stress-related genes, including *GmERD1* and *GmETR2* (Figure 6C), and the promoters of biotic stress-related genes, such as *GmETR2, GmABI4, GmERF7, GmETR1, GmNPR3* and *GmPR1*, which were also related to ETH (*GmETR1, GmETR2, GmERF7*), ABA (*GmABI4*), JA (*GmNPR3*) and SA (*GmPR1*) signaling pathways, respectively (Figure 6D). Moreover, three target genes (*GmETR1, GmERD1* and *GmETR2*) with the most enrichment of *GmbZIP19* were chosen to verify the direct interaction between *GmbZIP19* and these genes using yeast one-hybrid. 70 bp original fragments (named as *GmETR1, GmERD1, GmETR2*) containing the cis-element were used as bait and cloned into the pABAi vector, while GmbZIP19 was used as a prey. As shown in Figure 6E, the yeast cells of original and mutational fragment were co-transformed with or without *GmbZIP19*. Results showed that, when bait vector transformed alone into yeast, they grew normally in screening medium (SD/-Ura/-Leu) but were inhibited by 200 ng/mL AbA (Aureobasidin A), and the bait vector co-transformed with GmbZIP19 could survive under 200 ng/mL, which suggested that GmbZIP19 could directly bind to the *GmETR1, GmERD1* and *GmETR2* promoters.

## 3. Discussion

The bZIP TF family is a plant-specific transcription factor family and is involved in diverse signaling pathways underlying biotic and abiotic stress responses and biological processes. Although the genome-wide analysis of the bZIP transcription factor family has been conducted in soybean [17] and there are several bZIP genes’ functions reported in plants [26,27], little is known about the functions of bZIP genes in soybean. In our study, *GmbZIP19* was isolated from full-length soybean cDNA. The multiple alignment analysis showed that *GmbZIP19* contains a typically conserved DNA binding domain (N-x7-R/K-x9) and a DOG1 structural domain of 79 amino acids among different plants (Figure S1). The subcellular localization indicated that GmbZIP19 was localized in the nucleus (Figure S2). Moreover, numerous stress-related cis-elements, including G-box, MYB, CGTCA-motif and TCA-motif were present in the 2000 bp promoter region of the *GmbZIP19* gene (Figure S3). The MYB transcription factor can combine with MYBRS and is involved in ABA signaling pathway and abiotic stress responses [28]. Some genes can confer abiotic tolerance through their G-box [29]. Such an abundance of stress responsive cis-elements in the *GmbZIP19* promoter suggested a potential role of this gene in stress responses. Therefore, in order to explore the potential functions of *GmbZIP19* in response to different stresses, we overexpressed *GmbZIP19* in *Arabidopsis* and revealed that *GmbZIP19-OE Arabidopsis* exhibited significantly increased resistance to *S. sclerotiorum* and *Pseudomonas syringae* (*pst.* DC3000) but sensitivity to abiotic stresses, including salinity and drought.

Plants have a wide range of mechanisms to cope with many stresses in their natural environments [30]. In terms of disease defense, plants can recruit an inducible defense system to resist the infection of certain pathogens. Previous studies have shown that phytohormones, including ABA, SA, JA and ETH play crucial roles in biotic stress signaling following pathogen infection [31,32,33,34]. Overexpression of *ERF1* in *Arabidopsis* increased tolerances to some necrotrophic pathogens [35]. Overexpression of *GmKR3* in soybean enhanced virus tolerance through affecting ABA signaling [36]. In the current study, the expression of *GmbZIP19* in wild-type soybean was dramatically induced by *S. sclerotiorum* (Figure 1A), and GUS activity of *pGmbZIP19-GUS* transgenic leaves was also induced by inoculation of *S. sclerotiorum* (Figure 2G,H). Furthermore, the expression of *GmbZIP19* was activated by diverse hormones including JA, SA, ETH, ABA and BR (Figure 1B–F), indicating that *GmbZIP19* may be involved in pathogen defense which is related to different hormone signaling pathways. In addition, a significant upregulation in the expression levels of several marker genes responding to ABA, JA, SA and ETH was detected in the transgenic *GmbZIP19-OE* lines exposed to *S. sclerotiorum* compared to WT (Figure 3D), which further confirmed the involvement of phytohormone signaling in regulation of *GmbZIP19*-mediated pathogen resistance. *GmbZIP19* confers similar resistance to *pst*. DC3000 but only JA- and SA-dependent genes are detected to be functioning in *pst*. DC3000 resistance (Figure 3E,F). According to previous studies, PR2, a SA-responsive gene [37], is a key component in the SA defense signaling pathway. The increased expression of *PR2* in *GmbZIP19-OE* lines upon pathogen infection suggested that the SA signaling pathway may participate in pathogen tolerance of *GmbZIP19-OE* transgenic *Arabidopsis*. Moreover, the signaling pathways mediated by JA and ETH usually respond to necrotrophic pathogens, insects, herbivores and injury [38]. LOX4 and ERF1, which participate in the JA and ETH signaling pathways respectively, were upregulated in *GmbZIP19-OE* leaves upon *S. sclerotiorum* inoculation. The induced expression of the two marker genes by pathogen infection may indicate that JA and ETH are involved in the regulation of *GmbZIP19* to disease defense as well.

Previously, studies showed that various bZIP genes were involved in regulating plant response to abiotic stresses such as salt, drought and low temperature, etc. [13,39]. Overexpression of *TabZIP60* in wheat confers multiple abiotic stress tolerances [13], and overexpression of *TaWRKY2* could increase drought tolerance of transgenic wheat [40]. Different hormones are essential in the regulation of abiotic stress resistance. *OsbZIP72* serves as a positive regulator of ABA response and confers drought tolerance in transgenic rice [41]. Additionally, *OsJAZ1* alternates drought resistance by regulating JA and ABA signaling in rice [42]. In our study, the expression of *GmbZIP19* was reduced by salt and drought (Figure 1G,H), and the GUS activity of *pGmbZIP19-GUS* transgenic seedlings after abiotic stresses was also dramatically decreased (Figure 2E,F), suggesting that *GmbZIP19* is involved in abiotic response. Furthermore, our study showed that the salt and drought tolerance were significantly decreased in *GmbZIP19-OE* transgenic *Arabidopsis* compared to WT (Figure 4A,B and Figure 5A,B). Moreover, stomatal closure was defective in *GmbZIP19-OE Arabidopsis* with salt or drought treatment (Figure 4C–E and Figure 5C–E). Previous studies indicated that stomatal closure was associated with the ABA signaling pathway and therefore affected drought tolerance [43,44]. It is possible that the drought and salt sensitivity of *GmbZIP19-OE* transgenic lines was due to the defective stomatal aperture (Figure 4C–E and Figure 5C–E). Because the transcription factors may interact with the cis-elements in the promoter regions of many abiotic stress-related genes, thus affecting the expression of these stress-related genes, resulting in defective abiotic stress tolerance [45], it is necessary to detect the expression levels of stress-related marker genes. In the current study, the decreased expression of *WRKY26*, which is reported to be induced by various abiotic stresses such as salt, heat and cold treatment [46], directly confirmed the sensitivity of *GmbZIP19-OE* transgenic plants to abiotic stresses. In addition, the hormone-related marker genes involved in ABA, SA, JA and ETH signaling pathways were expressed at lower levels in *GmbZIP19-OE* plants than in the WT after salt or drought treatment (Figure 4F and Figure 5F), demonstrating that the hormone-related marker genes are involved in the abiotic stress tolerance. These results suggested that *GmbZIP19* affects the abiotic tolerances by being involved in the hormone responsive pathways.

Although *GmbZIP19-OE* plants were more sensitive to exogenous hormones than wild-type (Figure S4), the expression of *GmbZIP19* was induced by ABA, SA, JA and ETH (Figure 1B–E). The results implied the interlink between phytohormone signaling and *GmbZIP19*. ChIP-qPCR analysis showed that *GmbZIP19* could bind to the promoter of many hormone-associated stress-related genes (Figure 6C,D). The yeast one-hybrid assay confirmed the result (Figure 6E). These findings further confirmed that phytohormone signaling pathways are involved in *GmbZIP19* regulated biological processes.

In animals, there are several TFs that serve either as transcriptional activators or repressors [47]. In plants, few transcription factors with dual functions have been characterized. In *Arabidopsis*, *AtWRKY33* can activate the expression of *CYP71A13* and *PAD3*, while transcript levels of *NCED3* and *NCED5* increased simultaneously in the *wrky33* mutant after *B. cinerea* infections, suggesting that it acts as a repressor as well [48]. Also, ATAF2, a NAC domain transcription factor, acts as transcriptional activator or repressor dependent on promoter context [49]. In our study, *GmbZIP19* is a multi-functional TF that can regulate not only the disease defense but also the abiotic stress tolerance by participating in phytohormone signaling pathways. However, the more precise mechanism of *GmbZIP19* function and how *GmbZIP19* acts as an activator of biotic stress and a repressor of abiotic stress still need to be confirmed and the interaction of *GmbZIP19* with its homologous genes in *Arabidopsis* in stress responses continues to attract our attention.

In general, our study provides vital insights into the mechanism underlying the response of *Arabidopsis* and soybean to biotic and abiotic stresses and offer clues for brand new soybean designing, which can be more accessible to negative environments.

## 4. Material and Methods

### 4.1. Plant Materials and Growth Condition

*Arabidopsis thaliana* (*Columbia* ecotype) seeds were surface-sterilized with absolute ethyl alcohol for 5 min and 75% ethanol for 15 min, followed by washing three or four times with sterilized distilled water under aseptic conditions. The sterilized *Arabidopsis* seeds were placed on the Petri plates containing half-strength Murashige and Skoog (MS) medium. The plates were kept in the growth incubator for germination and development (22 °C, 16 h light/8 h dark). After seven days, the seedlings were transferred into soil and allowed to grow under control environmental conditions (22 °C, 16 h light/8 h dark).

Seeds of soybean (YC03-3) were sown in soil and grown at 25 °C with a 16 h light/8 h dark (normal condition). Two-week-old soybean plants were infected with *S. sclerotiorum* and treated with 100 μM JA, 1 mM SA, 100 μM ABA, 1 mM ETH, 100 μM BR, 100 mM NaCl for salt condition, 250 mM mannitol for drought condition and 4 °C (placed the soybean in a 4 °C refrigerator and used plant growth light to keep the light condition the same as other plants) for low temperature, respectively. Normal condition or control means there is no treatment with the *Arabidopsis* or soybean.

### 4.2. GmbZIP19 Sequence Analysis

The *GmbZIP19* (Glyma11G16050) cDNA was identified from Soybean cDNA libraries (https://phytozome.jgi.doe.gov/pz/portal.html) and the specific primers were designed, which are listed in Table S1. Homologous sequences of *GmbZIP19* in other species (*Arabidopsis* and rice) were downloaded from phytozome databases and sequence alignment was performed with DNAMAN.

### 4.3. The RNA Extraction and RT-qPCR Analysis

Total RNA used for the expression profile of *GmbZIP19* was isolated from leaves of wild-type soybean grown under normal growth conditions and different stress conditions, including salinity (NaCl), drought (mannitol), cold (4 °C), JA, ABA, SA, BR and ETH at 2, 6, 12, 24 and 48 h after treatment. In addition, total RNA of different tissues of wild-type soybean, including leaf, stem, root and inflorescence were isolated using a plant RNA extraction kit (OMEGA, China). During the extraction of RNA, RNase-free DNase (Promega, Madison, WI, USA) was used to avoid DNA contamination, and then the first-strand cDNA synthesis was carried out with approximately 1 mg RNA using the Transgene First Strand cDNA Synthesis Kit. The cDNAs were diluted five times to conduct RT-qPCR. Soybean *ACTIN* was used as reference gene (primers are listed in Table S2).

Total RNA used for transcription analyses of *GmbZIP19-OE Atabidopsis* was isolated from leaves of three-week-old WT and *GmbZIP19*-*OE Arabidopsis* plants under different stress conditions: 150 mM NaCl (0, 6, 12, 24, 48 h), 300 mM mannitol (0, 6, 12, 24, 48 h). Total RNA extraction and cDNA synthesis were carried out using methods described in the above sections. qPCR was performed to examine the relative expression levels of stress-related genes (the name and specific primers of these stress-related genes are listed in Table S2). In each case, all the experiments were repeated three times with all three technical replicates. Arabidopsis *HK2* was used as reference gene (primers are listed in Table S2).

To examine the specific expression of different materials, RT-qPCR was performed using SYBR Premix Ex Taq (TaKaRa, Toyoto, Japan). Reaction mixtures were prepared with a total volume of 20 μL each, containing: 1 μL of template, 8.2 μL of RNase-free water, 10 μL of 2× SYBR Premix and 0.4 μL of each specific primer according to the Bio-Rad Real-time PCR system (Foster city, CA, USA) and the SYBR Premix Ex II system (TakaRa Perfect Real Time). The RT-qPCR program was: 95 °C for 30 s, 40 cycles of 95 °C for 5 s and 60 °C for 34 s, and 95 °C for 15 s. The primers used for RT-qPCR are listed in Table S2. In each case, three technical replicates were performed for each of the three independent biological replicates. Only Ct values less than 40 were used to calculate correlation coefficients (R^2^ values) and amplification efficiencies (E) from the slope generated in Microsoft Excel 2013, based on the equation: E = [10^-(1/slope)^ − 1] × 100%. All PCR assays showed efficiency values between 95% and 110% [50].

### 4.4. Transformation and Generation of Transgenic Arabidopsis Plants and Stress Treatments

A 723 bp fragment of *GmbZIP19* (coding sequence) was amplified by PCR from the cDNA of wild-type soybean leaf with specific primers (shown in Table S1) and the promoter of *GmbZIP19* was cloned from the DNA of wild-type soybean with specific primers (shown in Table S1). The PCR products were cloned into pENTER/D-TOPO vector (Invitrogen) and then the recombinants were further fused into the destination vector pGWB605 and pGWB633 vector respectively, using Gateway LR Clonase II enzyme mix (Invitrogen). The 35S:*GmbZIP19*-GFP and 35S:*pGmbZIP19*-GUS recombinant vector was transformed into wild-type *Arabidopsis* (Columbia ecotype) using the *Agrobacterium*-mediated floral dip method [51]. Then, one-week-old T_1_ generation seedlings were sprayed with 0.4 mg/mL basta for selection of positively transformed plants and surviving seedlings were subsequently transferred to soil. The homozygous T_1_ generation seeds were harvested individually to obtain T_2_ generation seeds of different lines.

WT and transgenic *Arabidopsis* seeds were harvested at the same time. For salt, drought, ABA, JA and ETH, *GmbZIP19-OE Arabidopsis* and wild-type seeds were germinated on different types of 1/2 MS medium supplemented with 150 and 200 mM NaCl, 300 and 400 mM mannitol, 0.5 μM ABA, 200 μM JA and 450 μM ETH, respectively. In addition, seeds of *pGmbZIP19*-*GUS Arabidopsis* were planted on 1/2 MS medium with 150 mM NaCl and 300 mM mannitol, respectively. We used 1/2 MS medium without any supplementation as a control to germinate WT and transgenic *Arabidopsis* seeds. Plates were placed under controlled environmental conditions (22 ℃) for 4–7 days. The germination rate, root length and fresh weight of wild-type and transgenic *Arabidopsis* were measured and statistically analyzed. Three independent biological replicates were performed, and Student’s *t*-test was used for statistical analysis.

### 4.5. Subcellular Localization of GmbZIP19

The recombinant vector 35S-*GmbZIP19*-GFP was used for the subcellular localization study. To confirm the localization of GmbZIP19 fusion protein in cells, the 35S-*GmbZIP19*-GFP and 35S-GFP (vector control) recombinant fused plasmids were transformed into tobacco leaf cells using *Agrobacterium tumefaciens* strain GV3101.Transfected tobacco leaves were incubated in a greenhouse at 22 °C for more than 36 h. The fluorescence signals were monitored using confocal laser-scanning microscopy (SP5, Leica, Germany) with GFP fluorescence detection at 488 nm [52].

### 4.6. GUS Staining

Inflorescence samples were fixed in prechilled 90% acetone for 20 min and washed with distilled water. After brief vacuum infiltration, the inflorescences were incubated in β-glucuronidase (GUS) staining buffer overnight at 37 °C. After being cleared in 20% lactic acid/20% glycerol solution, the inflorescences were observed under a Leica (M205 FA) microscope.

### 4.7. S. sclerotiorum Infection and Staining

Three-week-old plants of WT and *GmbZIP19-OE Arabidopsis* were inoculated with *S. sclerotiorum.* The similar detached leaves of WT and *GmbZIP19*-*OE* lines are inoculated with the same size of *S. sclerotiorum*. The inoculated leaves were collected at 12 h post-infection. The inoculated leaves were stained with 1 mg/mL DAB (diaminobenzidin) for 8 h and then boiled in 75% ethanol for approximately 10 min to decolorize. The detailed methods of inoculation and disease scoring were the same as those described previously [53].

To verify the expression profile of stress-related genes under pathogen stress, we inoculated *S. sclerotiorum* to the three-week-old leaves and isolated inoculated leaves after 0, 6, and 12 h for total RNA extraction for qPCR (the stress-related genes are: *AtPR2, AtABI5, AtLOX4* and *AtERF1* and specific primers are listed in Table S2).

### 4.8. Pseudomonas Syringae Infection and Staining

*Pst* DC3000 was grown overnight in King’s B (KB) liquid medium containing 50 μg/mL rifampicin. The medium with bacteria was centrifuged at 2800 rpm for 10 min to collect bacteria. The pellet was resuspended in 10 mM MgCl_2_ and diluted to get OD_600_ = 0.001–0.002 for plant inoculation. Three-week-old WT and *GmbZIP19-OE Arabidopsis* plants were vacuum-infiltrated with suspension of *Pseudomonas syringae.* The inoculated leaves were sprayed with water and kept in a sealed container to ensure high humidity and the disease progress was observed daily [54]. Inoculated leaves were collected after 48 h post inoculation and stained with trypan blue, as mentioned below (trypan blue staining solution contains: 10 mL lactic acid, 10 mL glycerol, 10 g phenol, 10 mL distilled water, 40 mL 75% ethanol and 2 mL trypan blue). The leaves were boiled for 5 min in the trypan blue staining solution and then decolorized in chloral hydrate (0.5 g chloral hydrate dissolved in 1 mL of 75% ethanol) for at least 30 min and then viewed under a microscope with bright light [53].

### 4.9. Stomatal Aperture Observation under Different Stresses

Four-week-old WT and *GmbZIP19-OE Arabidopsis* were used to observe and measure the stomatal aperture conditions. Leaves of similar size, age and side branches of stems were selected to compare stomatal aperture. At first, the leaves and stems were infused in stomatal solution containing: 50 mM CaCl_2_, 10 mM MES, 5 mM KCl, pH 6.15 and exposed to light for approximately 2 h to ensure all the stomatal had opened. Subsequently, 150 mM NaCl and 300 mM mannitol were added to the solutions to induce salt and drought stress conditions, respectively. After 2 h of treatments, leaves and stems of WT and *GmbZIP19-OE Arabidopsis* were blended and observed with a digital microscope (Leica). The length and width of stomata were measured using image analysis (Adobe photoshop CC 2019) computer software [55].

To confirm the phenotype and quantitative result, we examined the expression levels of marker genes of stomatal movement and closure using *GsmbZIP19-OE* plants under salt or drought treatment. The stomatal movement marker genes are: *WRKY54* and *WRKY70*, the primers are listed in Table S2.

### 4.10. Transient Expression of GmbZIP19 and Chromatin Immunoprecipitation

Two-week-old soybean cultivar Huachun6 (YC03-3) was used to achieve the transient expression of *GmbZIP19*. The *Agrobacterium* containing recombinant vector 35S-*GmbZIP19*-GFP and 35S-GFP were grown overnight in LB liquid medium containing 50 μg/mL of spectinomycin and rifampicin. The media were centrifuged at 4000 rpm for 10 min. Pellets were resuspended in 3% sugar aqueous solution (*M/V*) with 0.1% silweet-77 (*V/V*). The leaves of soybean were infiltrated with *Agrobacterium* solutions. Two days after inoculation, the GFP signal of inoculated leaves was observed with a Leica TCS SP8X DLS confocal laser scanning microscope to confirm that the transient expression had been achieved successfully. The qPCR was performed by primers in Table S3 with *GmbZIP19* transient expressing soybean. Three technical replicates were performed for each of the three independent biological replicates.

For each chromatin immunoprecipitation (ChIP) experiment, 4 g of leaves were used. Crosslinked chromatin was fragmented with 0.2 units of micrococcal nuclease in 1 mL of MNase digestion buffer (10 mM Tris-HCl (pH 8.0), 50 mM NaCl, 1 mM-mercaptoethanol, 0.1% NP40, 1 mM CaCl_2_, and 1× protease inhibitor cocktail). Digestion was stopped using 5 mM EDTA. ChIP was performed using a GFP polyclonal antibody. Relative enrichment of associated DNA fragments was analyzed by qPCR. All oligonucleotide sequences used in the ChIP experiments are given in Table S3. Each ChIP experiment was repeated twice, and the presented data are from one representative experiment. In each case, three technical replicates were performed for each of the three independent biological replicates.

### 4.11. Yeast One-Hybrid System

*GmbZIP19* CDS without stop codon was amplified and then integrated into pGADT7 by the In-fusion cloning technique (Clontch, Takara) to form a pGADT7-GmbZIP19 effect vector. At the same time, the predicted binding sites were synthesized by DNA synthesis technology (the primers are listed in Table S4), and cloned into pABAi vector by In-fusion cloning technology to form pABAi-GmETR1, pABAi-GmETR2, pABAi-GmERD1, pABAi-GmNPR3 and pABAi-GmPR1 bait report vectors, respectively.

Yeast one-hybrid was carried out according to instructions provided by Clontech (Takara). Bait was transformed into Y1H gold yeast strain and cultured on SD/-Ura medium with or without 200 ng/mL Aureobasidin A (AbA) for 3 days. In addition, the yeast cells co-transformed by prey and bait were cultured on SD/-Leu medium containing 200 ng/mL AbA for 3 days.

### 4.12. Statistics Analysis

All experiments were carried out with three biological replicates and three technical replicates per biological replicate, and the data were shown as means ± standard errors (SD; *n* = 3). Asterisks indicate significant differences for the indicated comparisons based on a Student’s *t*-test (** *p* < 0.01; * *p* < 0.05). Three biological replicates were used for each of the genotypes (GUS staining, biotic and abiotic phenotype, stomatal aperture, DAB and trypan blue staining), including the wild-type and *GmbZIP19-OE-15*, *GmbZIP19-OE-16* and *GmbZIP19-OE-26* transgenic lines. For relative lesion area measurement, five biological replicates were performed for each line. For seed germination, root length and fresh weight measurement, more than ten biological replicates were performed. Asterisks indicate significant differences for the indicated comparisons based on a Student’s *t*-test (** *p* < 0.01; * *p* < 0.05).

## 5. Conclusions

In this study, we cloned and characterized soybean *GmbZIP19*. Overexpression of *GmbZIP19* in *Arabidopsis* resulted in increased tolerance to pathogen and decreased tolerance to drought and salt stress. Our results indicated that *GmbZIP19* increased plant pathogen tolerance and suppressed salt and drought stress response in association with plant hormones. Our findings obtained interesting basic scientific results that may shed light on the role of *GmbZIP19* TF in biotic and abiotic stress response which might be crucial for developing environmental stress-resistant soybean varieties.

## Supplementary Materials

Supplementary materials can be found at https://www.mdpi.com/1422-0067/21/13/4701/s1.
