# Supplementary figures and images for: A Soybean bZIP Transcription Factor GmbZIP19 Confers Multiple Biotic and Abiotic Stress Responses in Plant
Source: Int J Mol Sci. 2020 Jul 1;21(13):4701. doi: 10.3390/ijms21134701 (PMC7369738; doi:10.3390/ijms21134701)

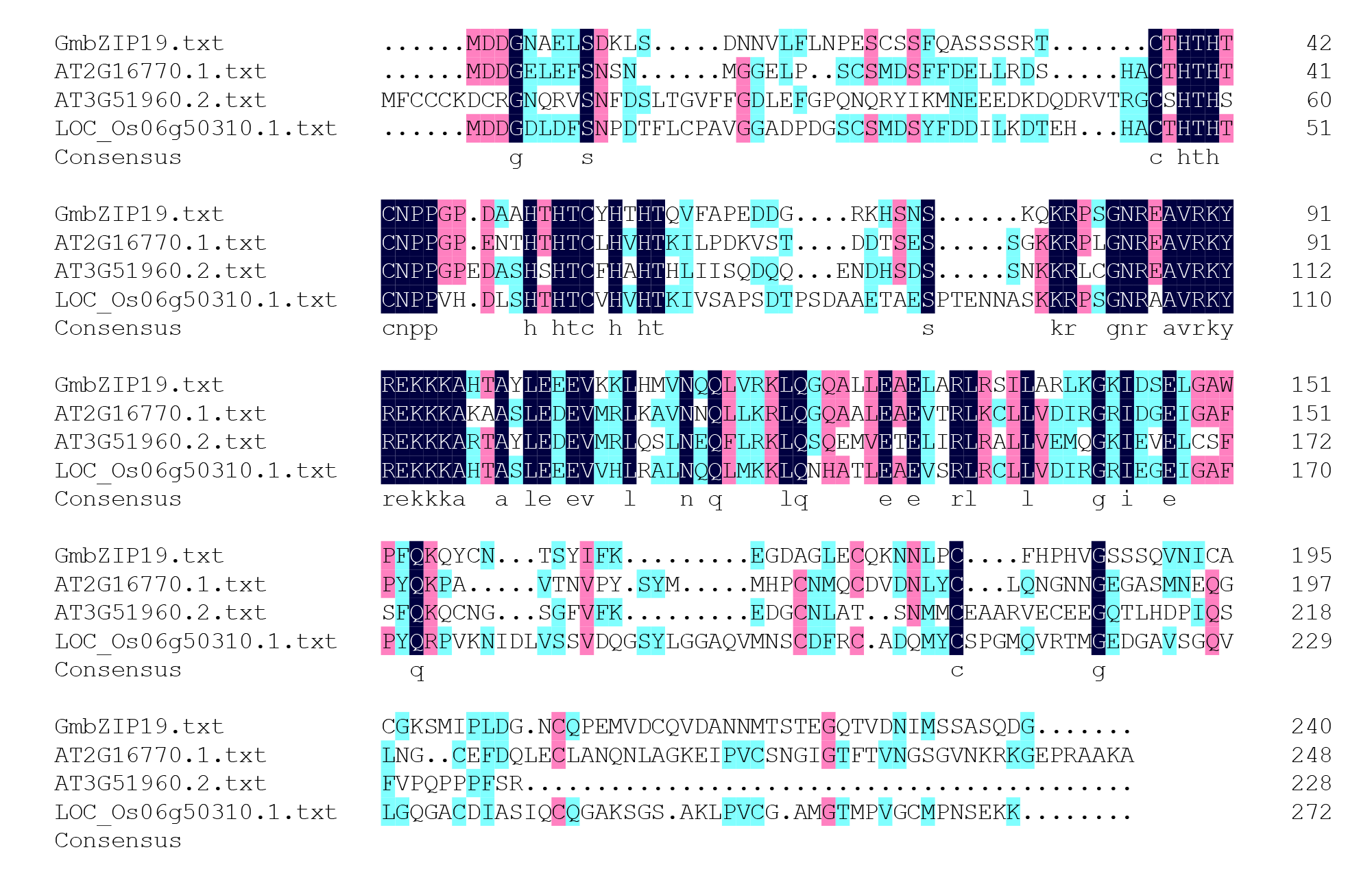

Supplement: Supplementary file 1 [file ijms-21-04701-s001.zip › GmbZIP19-supplementary files-update/GmbZIP19-figure S1.tif]

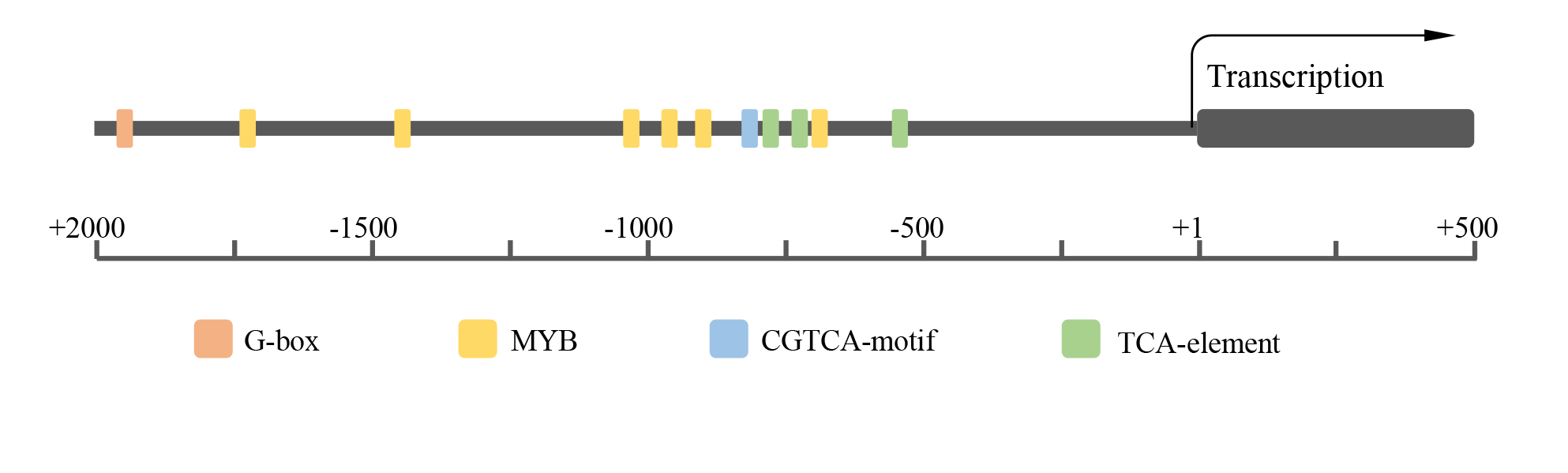

Supplement: Supplementary file 1 [file ijms-21-04701-s001.zip › GmbZIP19-supplementary files-update/GmbZIP19-figure S3.tif]

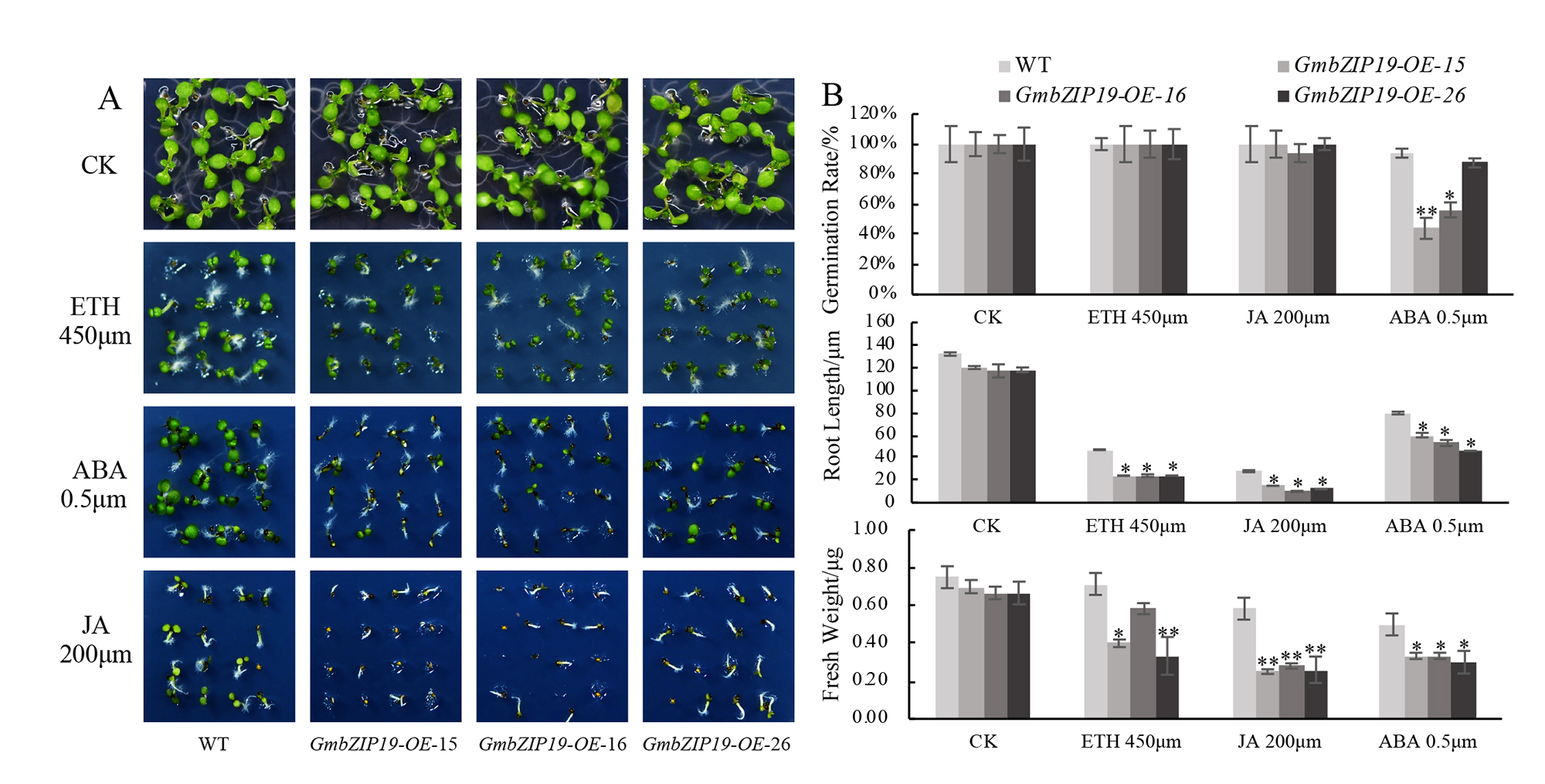

Supplement: Supplementary file 1 [file ijms-21-04701-s001.zip › GmbZIP19-supplementary files-update/GmbZIP19-figure-S4.jpg]
